# Cheilitis Glandularis: A Case Report of an Unusual Occurrence

**DOI:** 10.7759/cureus.99617

**Published:** 2025-12-19

**Authors:** Ashalata Gannepalli, Manvitha Juttukonda, Priyanka Venkatayogi, Sanjay Reddy Podduturi

**Affiliations:** 1 Oral and Maxillofacial Pathology and Oral Microbiology, Panineeya Institute of Dental Sciences and Research Centre, Hyderabad, IND; 2 Oral and Maxillofacial Pathology and Oral Microbiology, Sri Balaji Dental College and Hospital, Hyderabad, IND; 3 Oral and Maxillofacial Pathology and Oral Microbiology, Clove Dental Clinic, Hyderabad, IND; 4 Oral Medicine and Radiology, Denta On’e Advanced Dental Care Centre, Hyderabad, IND

**Keywords:** actinic cheilitis, cheilitis glandularis, igg4, lip, steroid therapy, uv exposure

## Abstract

Cheilitis glandularis (CG) is a rare chronic inflammatory condition of unknown etiology affecting the minor salivary glands of the lips. It presents as redness and dilatation of the ostia of minor salivary glands on the vermilion border, with variable degrees of macrocheilia and eversion of the lower lip.

A 36-year-old male patient had a chief complaint of burning sensation, with persistent swelling of the lower lip for two years and gradual appearance of ulcerations, crustations and intermittent oozing of liquid. Being a shepherd by occupation, he was constantly exposed to sunlight, suggesting the role of actinic radiation. The blood count parameters were normal with slightly raised ESR. No antibodies were detected in the antibody profile. On biopsy, histological evaluation showed prominent lympho-plasmacytic (Ig G4+++ plasma cells) infiltrate, atrophy of salivary acini, ductal ectasia, and sclerosis of collagen. The patient was treated with systemic, perilesional steroid injections and a topical immunosuppressant.

Cheilitis glandularis may mimic many other clinical conditions, hence thorough investigations are required to establish correct diagnosis and suitable care. The case highlights the actinic radiation-mediated inflammatory process as etiopathogenesis in such lesions.

## Introduction

The anatomically prominent location of the lips results in maximal exposure to ultraviolet (UV) radiation, food, and tobacco, among other factors that can cause substantial morbidity and lower self-esteem. Inflammatory disorders of the lips affecting the vermillion are commonly termed cheilitis, which ranges from actinic cheilitis, contact cheilitis, granulomatous cheilitis, plasma cell cheilitis, etc. It is also seen in various precancerous lesions, viz., leukoplakia, benign and malignant neoplasms, autoimmune disorders, systemic diseases, nutritional deficiencies, and infections. They are related to varying etiopathogenic and precipitating factors, which can present a diagnostic dilemma for the dental practitioners [1,2]. Among these, cheilitis glandularis (CG) is one such condition that is a specific subtype of stomatitis glandularis, producing lesions solely on the lips (predominantly lower) characterized by varying degrees of macrocheilia and mucopurulent exudate from the ductal orifices of the labial minor salivary glands [3,4]. In 1870, von Volkmann introduced the term cheilitis glandularis. According to one systematic review (2022), mean age of its incidence was 40.9 years, with males and light-skinned individuals affected greatly and a few women and pediatric cases were also reported [4,5]. Common clinical manifestations are swelling, erythema, eversion of the lip, dilated ductal openings, exudate, ulcers, induration, crust, dryness, protrusion, nodules, papules, cracks, and enlargement of the lip. The majority of cases reported were of simple type and association of CG with squamous cell carcinoma (SCC), lichen planus, leukoplakia, double lip, papillary cystadenoma, actinic cheilitis and mucous cyst were also noted. Though surgical treatment was indicated in a higher percentage of cases, a conservative approach with topical medications did result in resolution in a considerable number of cases [5]. The exact cause of CG is unknown, with several reports suggesting prolonged exposure to sunlight in the pathogenesis [6,7]. This condition presents with many overlapping features in common with other lip lesions. To aid accurate diagnosis, Reiter et al. proposed a set of diagnostic criteria that integrates both clinical presentation and histological findings. Clinically, the condition is characterized by the involvement of more than one minor salivary gland and by the presence of mucoid and/or purulent discharge from the apertures of the involved minor salivary glands. Histologically, the diagnosis is supported by the observation of sialectasia, or dilation of the salivary gland ducts, chronic inflammation, mucous/oncocytic metaplasia (ducts and/or acini), and mucin in ducts, reinforcing the importance of biopsy in such lesions [6]. Here, we present a case of cheilitis glandularis of the lower lip treated with systemic, perilesional corticosteroids and topical steroid therapy.

## Case presentation

**Figure 1:**
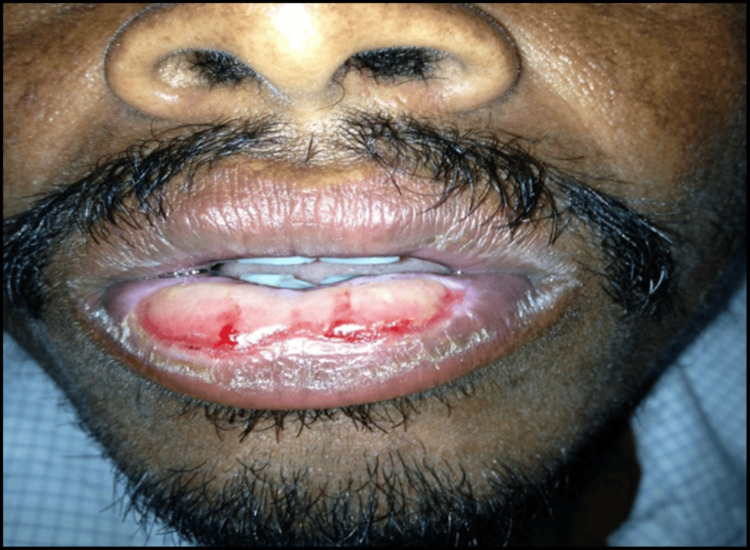
Diffuse swelling of the lower lip showing ulceration and crustations, with obliteration of vermilion border.

A 36-year-old male reported with a chief complaint of persistent swelling, pain and burning sensation of the lower lip for about two years to the clinic. Previous history revealed that he initially noticed two erythematous swellings on each side of the lower lip, which then gradually progressed with surface ulcerations. He consulted many doctors in and around his village, with on and off antibiotic therapy, but the lesion prevailed. For six months the swellings increased with severe burning sensation, pain, ulcerations and discharge of watery fluid, which he thought was pus. The patient had no known allergies and no habits of lip biting or lip sucking, but was constantly exposed to sunlight because of his occupation as a shepherd. The personal and family history was non-contributory. Clinical examination revealed a well-developed, moderately nourished man with poor oral hygiene. On extraoral examination, his lower lip was enlarged and grossly everted. Examination of the lesion proper revealed two oblong-shaped swellings of 2.0x1.5 cm in size on either side of the lip from the labial mucosa extending onto the vermilion border. The surface was erythematous, moist with thinning of mucosa and ulcerations, sloughing in the center with yellowish crustations. The surrounding area was dry and scaly. On palpation it was multinodular and soft in consistency with indiscernible indurated borders (Figure 1).

A working diagnosis of hypersensitivity reaction was proposed with differential diagnosis of cheilitis glandularis, cheilitis granulomatosa, actinic cheilitis, and autoimmune disorder. He was given 25mg antihistamine hydroxyzine three times daily for one week, and amoxicillin clavulanic acid 375mg three times daily, application of topical anesthetic-benzocaine gel, three times a day for pain relief just before meals and was advised laboratory investigations such as complete blood picture, random blood sugar (RBS), differential count, erythrocyte sedimentation rate (ESR), and antibody profile. After five days of review there was not much improvement clinically and blood count parameters were normal with slightly elevated ESR. There were no antibodies detected in basic antibody profile for autoimmune disorders (antinuclear antibody (ANA)-IgG, Anti-dsDNA, Anti-Ro/SSA, Anti-La/SSB, Anti-Sm, Anti-ribonucleoprotein (RNP)) (Table 1).

**Table 1:** Details of laboratory investigations done for the patient Complete Blood Picture (CBP), Red Blood Cell (RBC), White Blood Cell (WBC), Mean Corpuscular Volume (MCV), Mean Corpuscular Hemoglobin (MCH), Mean Corpuscular Hemoglobin Concentration (MCHC), Red Blood Cell Distribution Width (RDW), Erythrocyte Sedimentation Rate (ESR), Random Blood Sugar (RBS), Antinuclear Antibody Immunoglobin G by Immunofluorescence (ANA-Ig G (IF), Rheumatoid factor (RA factor), Anti-double stranded DNA (Anti-ds DNA), Anti-Sjogren's-syndrome related antigen A & B autoantibody (Anti-Ro/SSA & Anti La/SSB), Anti Smith (Anti-Sm), Anti Ribonucleoprotein (Anti-RNP), Not applicable (NA)

TEST NAME	RESULT	UNIT	BIOLOGICAL REFERENCE RANGE
CBP: Hemoglobin	15.3	g/dL	14-18
Total RBC count	5.07	X10^6 /µL	4.5-5.5
Packed Cell Volume / Hematocrit	47.7	%	40-54
MCV	94.1	fL	83.0-101.0
MCH	30.2	pq	27.0-32.0
MCHC	32.1	gm/dL	31.5-34.5
RDW	13.3	%	11.6-14.0
Total Leucocytes (WBC) Count	7.28	X10^3^/µL	4.0-10.
Differential Count: Neutrophils	58.7	%	40-80
Lymphocytes	34.9	%	20-40
Eosinophils	2.3	%	0.0-6.0
Monocytes	3.3	%	0-10
Basophils	0.5	%	<2
Absolute Neutrophil Count	4.27	X10^3^/µL	2.0-7.0
Absolute Lymphocyte Count	2.54	X10^3^/µL	1.0-3.0
Absolute Monocyte Count	0.17	X10^3^/µL	0.2-1
Absolute Basophil Count	0.24	X10^3^/µL	0-0.1
Absolute Eosinophil Count	0.04	X10^3^/µL	0-0.5
Platelet Count	238	X10^3^/µL	150000-410000
Peripheral Smear: RBCs, WBCs, platelets	Normocytic normochromic Adequate Platelets appear adequate in smear	NA	NA
ESR	20	mm/hr	0-15
RBS	90	mg/dL	80-140
Antibody Profile: ANA-Ig G (IF)	Negative	NA	NA
ANA Basic profile - RA factor	Negative	NA	NA
Anti-ds DNA	Negative	NA	NA
Anti-Ro/SSA	Negative	NA	NA
Anti-La/SSB	Negative	NA	NA
Anti-Sm	Negative	NA	NA
Anti-RNP	Negative	NA	NA

**Figure 2:**
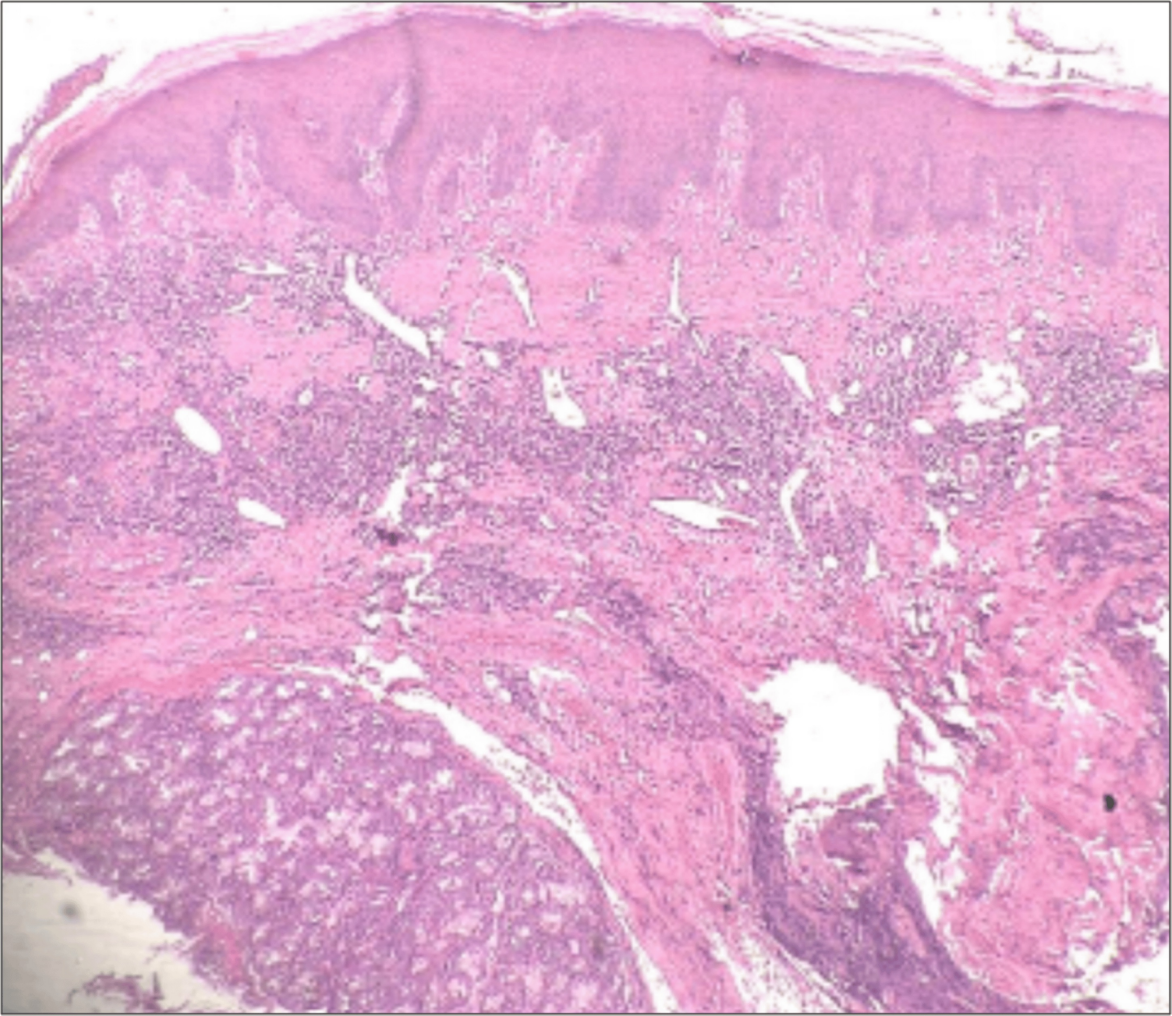
Photomicrograph (4x H&E-Hematoxylin & Eosin stain) showing surface epithelium with underlying connective tissue showing lymphoplasmacytic infiltrate around dilated blood vessels and salivary gland acini.

**Figure 3:**
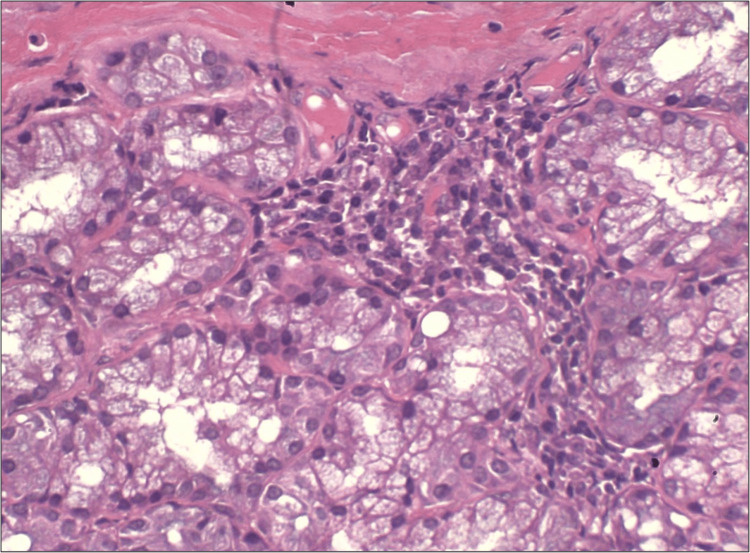
Photomicrograph (40x H&E) showing inflammatory cell infiltration of salivary gland acini.

**Figure 4:**
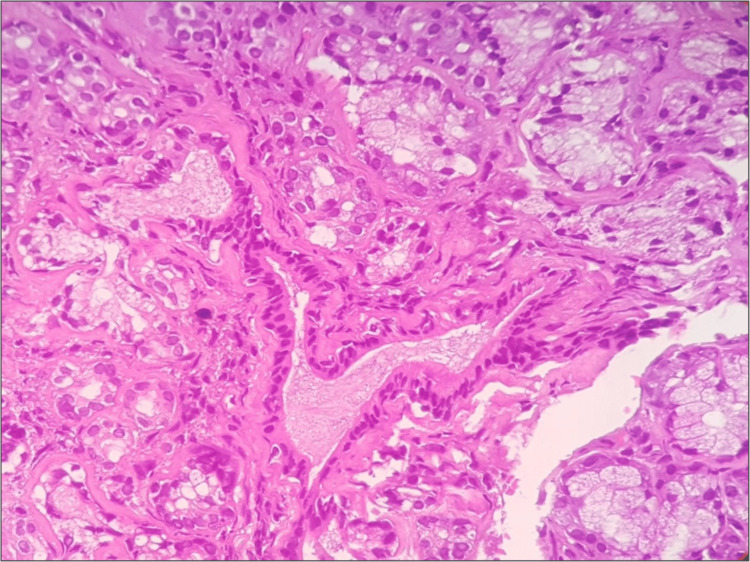
Photomicrograph (10x H&E) showing large dilated ducts with mucin pooling.

**Figure 5:**
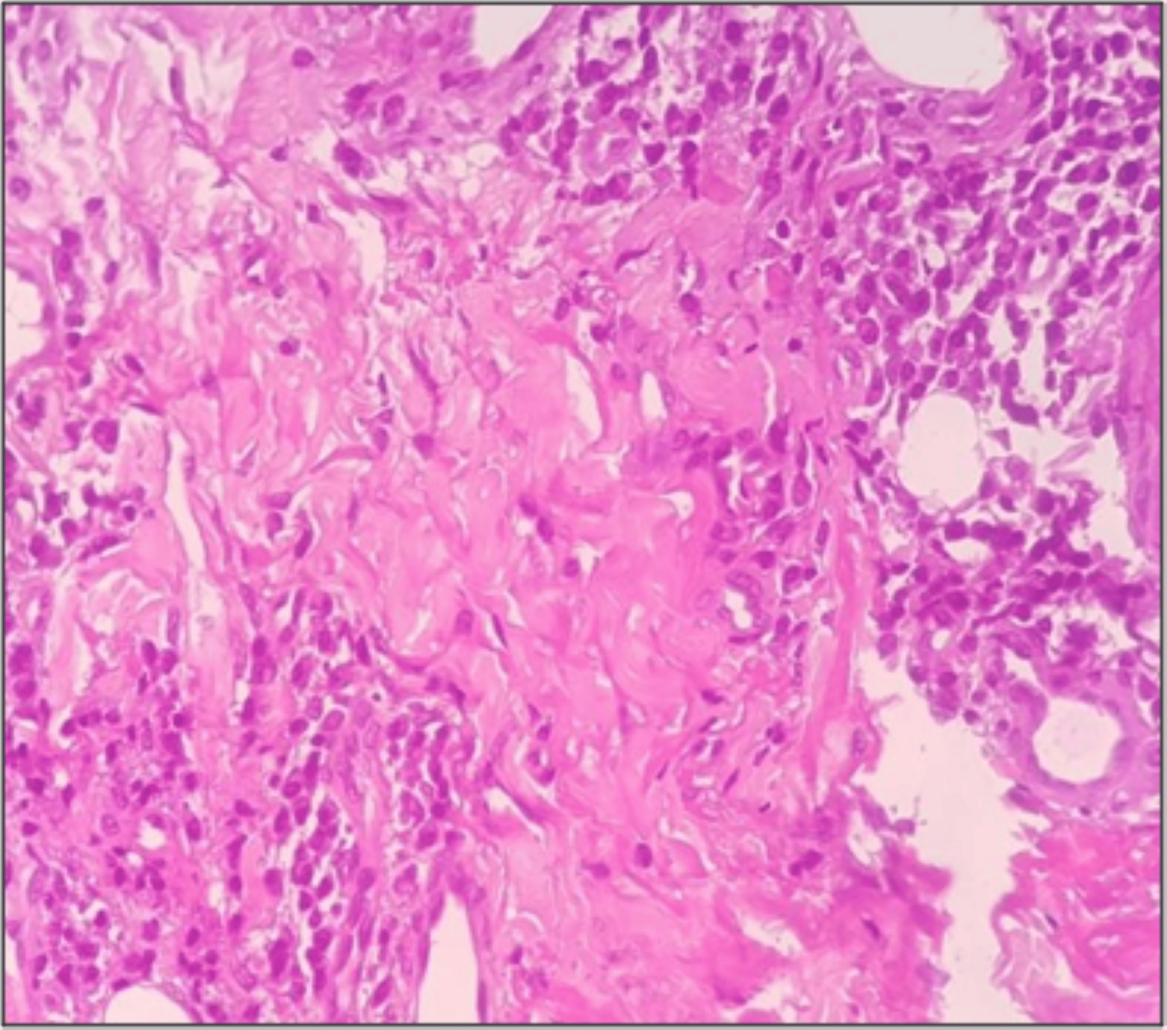
Photomicrograph (40x H&E) showing storiform sclerosis.

**Figure 6:**
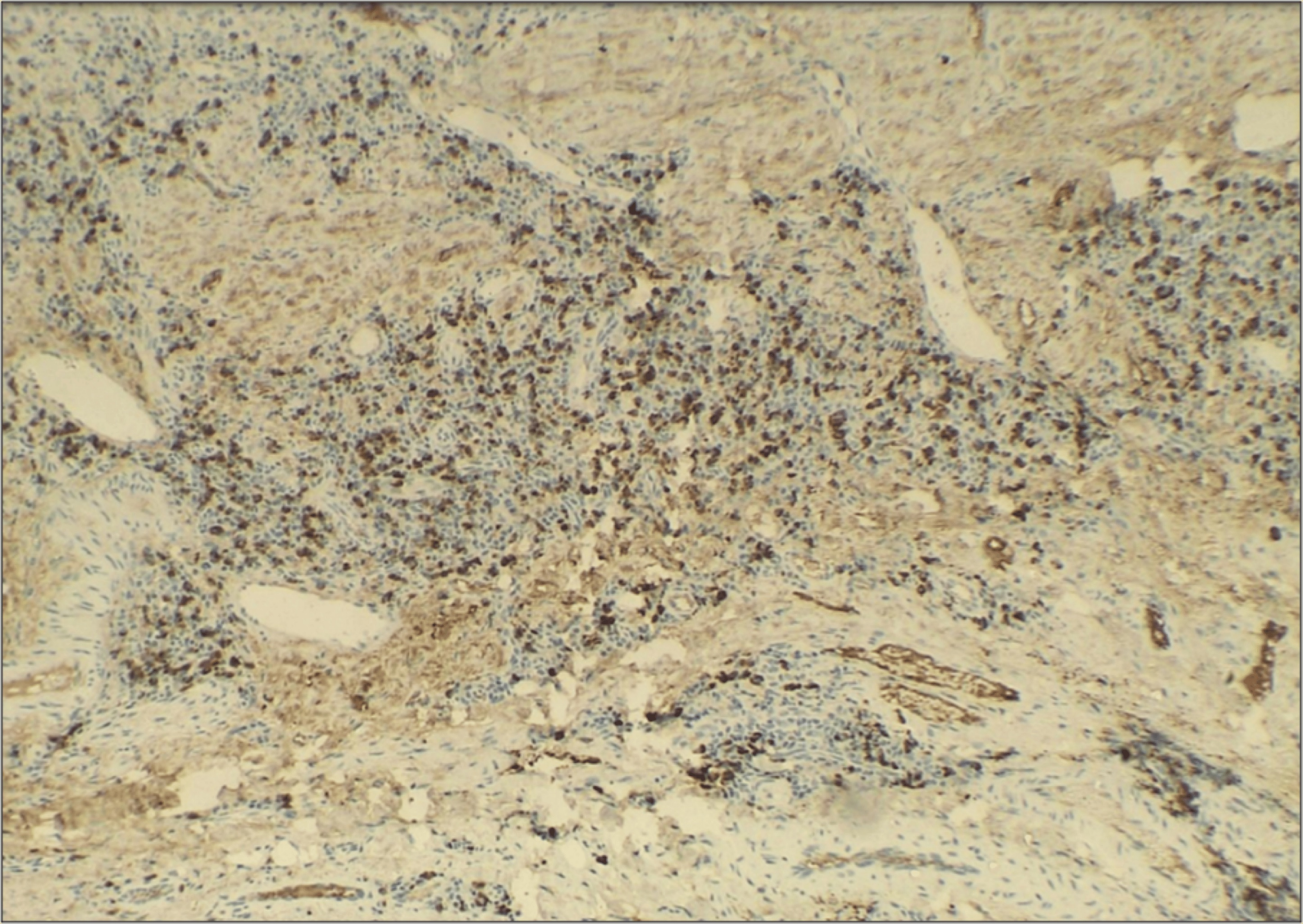
Photomicrograph (10x IHC) showing diffuse strong positivity as shown by plasma cells with IgG4.

Punch biopsy was done from the margin of the lesion and sent for histopathological examination to the department of oral pathology and microbiology. Histopathology displayed focal aggregates of chronic lymphoplasmacytic infiltration around dilated vascular channels, salivary gland acini and ducts (Figures 2, 3). Few areas showed atrophy of salivary gland with dilated ducts filled with mucin (Figure 4). The intervening stroma had sclerosis of collagen in a few areas and epithelium was hyperkeratotic with no dysplasia (Figure 5). There were no bacterial/or fungal colonies. Immunohistochemical evaluation with IgG4 (Anti-human IgG4 rabbit monoclonal antibody; BioGenex, Fremont, CA, USA) showed positivity (+++) in plasma cells (Figure 6). Based on clinical and histopathological findings, a diagnosis of cheilitis glandularis was made.

**Figure 7:**
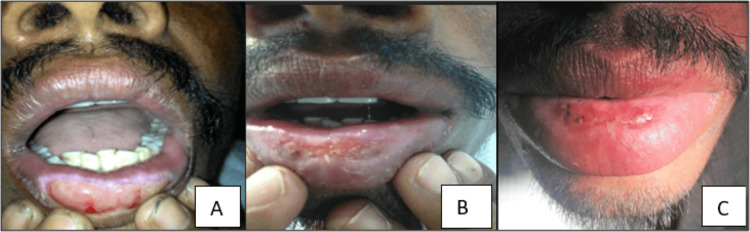
Resolution of lesion from periphery to center. A: first week after steroid therapy and peri-lesional injections, B: after one and a half month of treatment, C: after two months of treatment

The patient was administered oral systemic steroid 10mg prednisolone tablet early morning for 10 days, followed by alternate day 10 mg prednisolone tablet for two weeks. The submucosal injections of 1ml of betamethasone sodium phosphate (4mg/ml) with 1ml of 2% lignocaine hydrochloride were administered perilesionally. The combination was given for pain relief and also for its deposition over a large area. The sub-mucosal injections were administered perilesionally with a four-day interval for one month. The injections were discontinued after one month with only topical application of 0.1% triamcinolone ointment twice a day, but within a week there was a mild recurrence of oozing and crusting. The injections with a four-day interval were further continued perilesionally along with topical 0.1% triamcinolone ointment for one more month. The patient was reviewed every week and we noticed regression of symptoms and reduction in size of the lesion from the periphery. The patient was advised to continue with topical triamcinolone ointment for the next 15 days, use sunscreen lip balm (SPF30) twice a day, and continue follow-up for six months (Figure 7).

## Discussion

CG presents in three types: CG simplex, CG purulent superficialis and CG apostematosa (deep suppurative, CG suppurativa profunda, myxadenitis labialis) [6,7]. These are reported to be stages of a single progressive disorder which range from painless papules, indurated lip swellings, ulcerations to deep-seated infection with abscesses, sinus tracts and fistulas [7]. Our patient had shown involvement of labial mucosa extending onto the vermilion borders of the lower lip, with erythematous swellings that later progressed to ulcerations, which can have varying etiopathogenesis from infections, trauma, autoimmune, allergic reactions, and granulomas and can present a diagnostic and therapeutic challenge [6]. Immune-mediated diseases that mimic CG are erythema multiforme, pemphigus vulgaris, and allergic reactions that can present as swelling of the lips. Allergic reaction/angioedema usually resolves in 24-48 hrs and recurs again on antigenic stimulation. Immune-mediated disorders affecting only the lip are rare and on complete antibody profile, antibodies against ANA, RA, Anti-ds-DNA, Anti-Ro/SSA, Anti-La/SSB, Anti-Sm, Anti-RNP were not detected.

Factitious cheilitis is associated with the habit of lip sucking and biting characterized by crusting, ulcerations, presence of epithelial tags. Cheilitis granulomatosa shows similar clinical resemblance to CG, but has prominent non-caseating granulomas [6,8]. In this case, the patient gave history of prolonged exposure to sunlight with everted lip and ulcerations, and we performed biopsy to rule out actinic cheilitis or an underlying carcinoma. Clinically actinic cheilitis appears as pale areas, diffuse white plaques, atrophy of the lip and in later stages may cause erosion, ulcers, crusting, leading to alteration of epithelium and basophilic solar elastosis of collagen and elastic fibers [9]. CG and actinic cheilitis occurring on the lower lip in a female and CG with epithelial dysplasia have been reported [10,4]. CG has been considered a potential predisposing factor in the development of epithelial malignancy, but few authors assume it as an incidental finding. Nico et al. and Butt et al. presented cases of CG associated with SCC. Nico et al. suggested CG may be a secondary reactive change in salivary glands than a frank etiological factor for development of malignancy [11,12]. As actinic cheilitis has a 10-30% of malignant transformation of the lower lip, our case underscores the importance of histopathology in differentiating the above lesions and to rule out dysplasia or carcinoma, which was of major concern in the case [9]. With the advent of new diagnostic modalities, antibody profile plays an important role in identifying any underlying autoimmune and immune-mediated disorders.

The concept of IgG4-producing plasma cells is implicated in the pathogenesis. Reinhard E. Friedrich et al. stated it might be a variant of IgG4-related diseases [13]. Umehera et al. suggested diagnostic criteria for IgG4-related disorders, which includes ˃135mg/dl IgG titre, more than 40% cells showing positivity with IgG4 and histopathologically presence of storiform arrangement of collagen and sclerosis [14]. In order to establish association, we performed an IHC study using the IgG4 marker which showed positivity (+++) in the majority of plasma cells and in deeper connective tissue there was sclerosis with few areas of storiform collagen. Hence, CG might be an inflammatory reaction mediated by plasma cells to predisposing factors, actinic radiation being cause here rather than a primary salivary gland disease. As serum IgG4 levels were not done, the diagnostic relevance is less definitive to consider it as an IgG4-related disorder, which further needs to be evaluated in similar cases. 

Plasma cell cheilitis (PCC) is an idiopathic inflammatory disorder of lips which presents as a dark red or brownish patch or plaque, ulcerations, erosions, fissures in lower lip of elderly people and histopathologically shows hyperkeratosis, dyskeratosis, intercellular edema, vacuolar or liquefactive degeneration of dermo-epidermal junction, many plasma cells infiltrating in a band like shape in upper dermis or dense infiltration in entire dermis. The causative agent for PCC is also suggested to be solar damage as it is most common in the lower lip. The treatment consists of steroids and immunomodulatory drugs (viz. tacrolimus, pimecrolimus) [15]. Hence, solar damage might have variable clinical presentations manifest in different ways in different individuals. The histopathological features of CG are non-specific with a wide variety of possible clinical differential diagnosis and needs a clear clinico-pathologic correlation [6]. Thus, actinic cheilitis, PCC, immune-mediated disorders more so, epithelial dysplasia and any associated or superimposed bacterial or fungal etiology were also ruled out on contemplating steroid therapy.

The exact cause of CG is still unclear, naming multiple predisposing factors viz., smoking, poor oral hygiene, chronic exposure to sunlight, wind, atopy, diabetes mellitus, drug-induced xerostomia, a compromised immune system, local trauma, and even genetic predisposition and heredity [13,16,17]. A case of CG accompanied by Pseudomonas aeruginosa Infection was reported, highlighting the need for bacterial culture whenever the infection is suspected [17]. The role of sun exposure has been suggested; UV radiation can cause changes in the epithelium, which progresses to involve ductal orifices, then subsequent salivary retention and finally inflammation. Alternatively, macrophages, Langerhans cells can recognize photoallergens in skin, which present to effector T cells and then inflammatory reaction. A case with familial history in a pediatric patient was also reported, suggesting genetic predisposition [6,18]. Recent reports state that initially CG can display hyposalivation that produces an imbalance in aquaporins (AQPs) and alterations in the final composition of saliva, making it more viscous than normal saliva; upon that, certain external factors could worsen the condition, causing more inflammation and retrograde infection, resulting in mucopurulent discharge, implying actinic damage could be an aggravating factor, when the lesion is exposed to UV radiation [3]. 

Various treatment modalities have been described based on hisopathological analysis, which includes administration of antihistamines, antimicrobials if secondarily infected, intralesional steroids, topical immunosuppressants and in severe cases, surgery [3]. Surgical treatment options include vermilionectomy, surgical stripping, cryosurgery and laser therapy may be employed in some conditions [8]. Excellent prognosis was reported in a case by treating with intralesional triamcinolone acetonide (ILTAC) 40mg, topical tacrolimus alongside regular petroleum jelly application and photoprotection, stating this as a cost-effective therapeutic modality [19].

In our case, the initial treatment was an antihistamine for any allergic contactants along with antibiotics, suspecting the presence of an underlying bacterial/fungal infection. The topical anaesthetic agent was administered for symptomatic relief. After the final diagnosis of CG was made, surgical stripping was planned but the patient refused the surgical option. As the patient was in severe distress, we started oral intermediate-acting corticosteroid as well as peri-lesional submucosal injections of long-acting steroid simultaneously for about one month, which showed rapid resolution of the lesion, followed by application of a topical immunosuppressant. But after stoppage of intralesional steroid, there was a recurrence; therefore, submucosal injections as well as topical immunosuppressant were continued for about another month.

## Conclusions

Cheilitis glandularis, a rarely encountered entity, may represent an actinic-mediated inflammatory disorder and can pose a diagnostic and therapeutic challenge. Clinically it may mimic many other conditions, hence thorough investigation is required to establish the correct diagnosis. A history of constant sun exposure emphasizes for a histopathological diagnosis to rule out underlying dysplasia for proper management.
